# Frequent Somatic Mutation in Adult Intestinal Stem Cells Drives Neoplasia and Genetic Mosaicism during Aging

**DOI:** 10.1016/j.stem.2015.09.016

**Published:** 2015-12-03

**Authors:** Katarzyna Siudeja, Sonya Nassari, Louis Gervais, Patricia Skorski, Sonia Lameiras, Donato Stolfa, Maria Zande, Virginie Bernard, Thomas Rio Frio, Allison J. Bardin

**Affiliations:** 1 Institut Curie, 26 rue d’Ulm, F-75248 Paris, France; 2 CNRS UMR3215, F-75248 Paris, France; 3 INSERM U934, F-75248 Paris, France; 4 Next-Generation Sequencing Platform, Institut Curie, Hôpital Curie, 8 rue Louis-Thuillier, 75248 Paris Cedex 05, France

## Abstract

Adult stem cells may acquire mutations that modify cellular behavior, leading to functional declines in homeostasis or providing a competitive advantage resulting in premalignancy. However, the frequency, phenotypic impact, and mechanisms underlying spontaneous mutagenesis during aging are unclear. Here, we report two mechanisms of genome instability in adult *Drosophila* intestinal stem cells (ISCs) that cause phenotypic alterations in the aging intestine. First, we found frequent loss of heterozygosity arising from mitotic homologous recombination in ISCs that results in genetic mosaicism. Second, somatic deletion of DNA sequences and large structural rearrangements, resembling those described in cancers and congenital diseases, frequently result in gene inactivation. Such modifications induced somatic inactivation of the X-linked tumor suppressor *Notch* in ISCs, leading to spontaneous neoplasias in wild-type males. Together, our findings reveal frequent genomic modification in adult stem cells and show that somatic genetic mosaicism has important functional consequences on aging tissues.

## Highlights

• The aging *Drosophila* intestine is genetically mosaic
• Somatic recombination, genomic deletions, and rearrangements occur in aging ISCs
• Somatic inactivation of the tumor-suppressor *Notch* causes male-specific neoplasia

## Graphical Abstract

**Figure:**
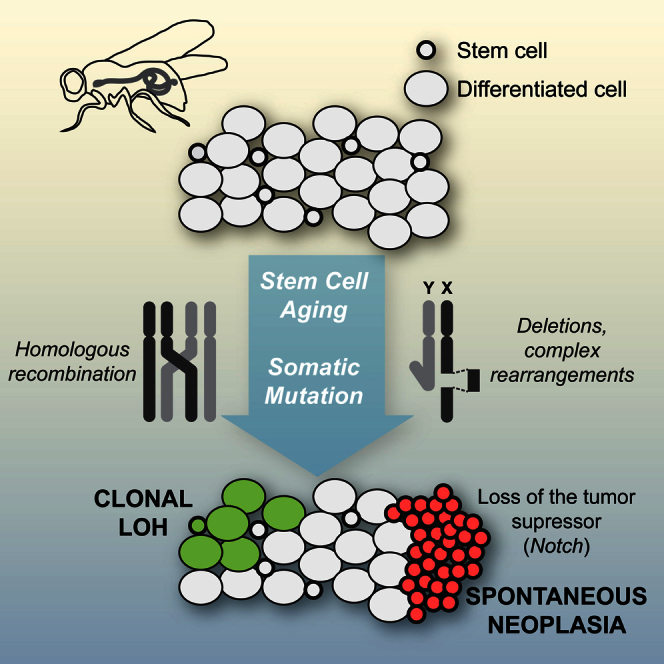

## Teaser

Bardin and colleagues show that aging *Drosophila* intestinal stem cells (ISCs) acquire frequent spontaneous mutations, including frequent loss of heterozygosity arising from homologous mitotic recombination that results in clonal mosaicism. They also show spontaneous gene deletions and chromosomal rearrangements in aging ISCs, which can promote neoplasia formation through inactivating *Notch*.

## Introduction

During aging, defects in stem cell function contribute to a decline in renewal and repair of adult tissues (Behrens et al., 2014). It has long been postulated that the accumulation of somatic mutations might contribute to cellular aging (Failla, 1958, Szilard, 1959). In support of this notion, induced DNA damage or mutations affecting DNA repair components can mimic some of the effects of aging on stem cells (Inomata et al., 2009, Nijnik et al., 2007, Rossi et al., 2007). Moreover, γ-H2AX foci and double-strand break accumulation in aging stem cells is consistent with DNA damage occurrence (Beerman et al., 2014, Rossi et al., 2007, Rübe et al., 2011). This, however, remains controversial as recent work indicates that γ-H2AX accumulation is due not to DNA damage per se but to an age-dependent increase in replication stress, which in turn, leads to stem cell functional decline (Flach et al., 2014). Furthermore, it remains unaddressed whether such signs of genomic instability result in somatic mutations sufficient to contribute to age-related functional decline.

While somatic genetic mutation has been well documented in cancers, its occurrence in and ultimate effects on healthy tissues is less well defined. Recent analysis of healthy humans has demonstrated that somatic copy number variation can arise in the blood (Jacobs et al., 2012, Laurie et al., 2012), colon (Hsieh et al., 2013), skin (Martincorena et al., 2015), and other adult tissues (McConnell et al., 2013, O’Huallachain et al., 2012). In addition, nucleotide variants have been shown to arise during development and adult life in mouse and human tissues (Behjati et al., 2014, Lodato et al., 2015, Martincorena et al., 2015). In *Drosophila*, classic studies from Stern (Stern, 1936) demonstrated that rare mitotic crossover events leading to loss of heterozygosity (LOH) occur in somatic tissue. In addition, a *LacZ* assay suggested an increase in spontaneous mutation during aging in flies (Garcia et al., 2007, Garcia et al., 2010). However, despite these findings, the frequency, mechanisms, and phenotypic consequences of somatic genetic variation on adult stem cells and tissues remain unclear and need to be addressed.

*Drosophila* is a well-established model for studying organismal aging and adult intestinal stem cells have become an important system to understand fundamental mechanisms controlling stem cell function. The *Drosophila* adult midgut is composed of around 10,000 cells renewed weekly by a population of approximately 1,000 multipotent intestinal stem cells (ISCs) (Micchelli and Perrimon, 2006, Ohlstein and Spradling, 2006). The ISC has a simple lineage, thought to lack transit-amplifying divisions, where ISCs are the primary, if not only, dividing cell type. In a young homeostatic midgut, ISCs divide rarely, whereas in an aged tissue increased activity of stress response pathways leads to enhanced stem cell proliferation and epithelial dysplasia (Biteau et al., 2008, Choi et al., 2008, Guo et al., 2014).

Here we demonstrate that somatic genetic variation is an important consequence of aging. We report a surprisingly high frequency of genetic variation in the aging *Drosophila* intestine and we decipher at least two mechanisms by which intestinal stem cells acquire mutations. We show that somatic recombination occurs frequently in aging ISCs, which leads to spontaneous inactivation of single copy transgenes or LOH. Furthermore, we identify homologous recombination-independent somatic DNA sequence deletions and large chromosomal rearrangements that lead to inactivation of the X-linked tumor suppressor gene *Notch* in wild-type males and have hallmarks of chromothriptic-like events recently described in cancers and congenital diseases. Somatic *Notch* inactivation, in turn, has direct consequences on tissue homeostasis as it leads to the formation of spontaneous male neoplasias.

## Results

### Somatic Recombination Drives Frequent Loss of Heterozygosity in Aging Stem Cells

**Figure 1:**
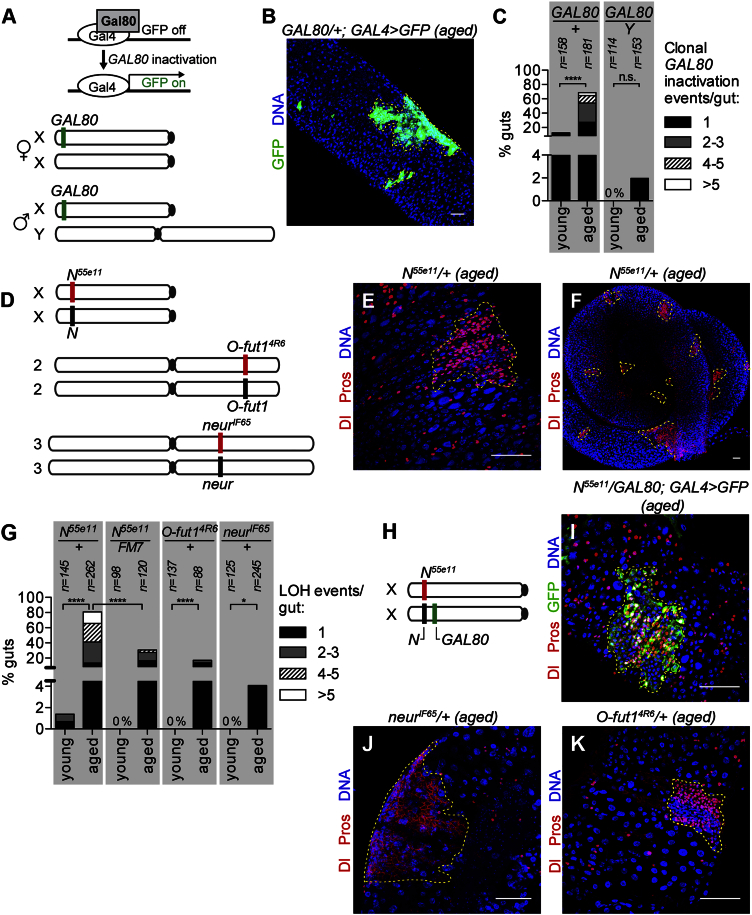
Somatic Recombination Contributes Strongly to Frequent Spontaneous Loss of Heterozygosity in Aging Fly Midguts (A) Inactivation of *GAL80* leads to Gal4-dependent GFP expression. A *GAL80* transgene at position 1E was used in females (XX) and males (XY). (B) Spontaneous GFP+ clones from an aged female midgut. (C) Frequency of GFP LOH events in young (0 to 1 week) and old (5 to 6 weeks) female (*GAL80*/+) and male (*GAL80*/Y) midguts. (D) Chromosomal locations of LOH markers *Notch* (*N*), *O-fut1*, and *neur*. (E) *Notch* LOH events in aged *N*^*55e11/+*^ midguts were identified by staining for Delta (Dl, cytoplasmic red) and Prospero (Pros, nuclear red). (F) A female *N*^*55e11/+*^ midgut with numerous LOH events. (G) Frequency of LOH events in young and aged female midguts heterozygous for *N*, *N^55e11^/ FM7* (balancer chromosome), *O-fut1*, and *neur*. (H) *N*^*55e11*^ at position 3C and *GAL80* at position 5B. (I) An LOH clone in an aged *N*^*55e11*^*/GAL80* midgut with *N* phenotype and *GAL80* inactivation. (J) Spontaneous *neur* LOH clone in an aged female *neur*^*IF65*^*/+* midgut. (K) Spontaneous *O-fut1* LOH clone in an aged *O-fut1*^*4R6*^*/+* midgut. LOH clones are outlined in yellow. Scale bars: 50 μm. ^∗^p < 0.05, ^∗∗∗∗^p < 0.0001; n.s., not significant (Fisher’s exact test, two-tailed). See also Figure S1 and Table S1.

To study somatic genome instability in adult intestinal stem cells, we first assessed the frequency of spontaneous inactivation of a single copy transgene inserted on the X chromosome at position 1E. The inactivation of the Gal4 repressor *GAL80* in ISCs leads to heritable, clonal, Gal4-driven GFP expression (Figure 1A). GFP-positive single cells as well as clonal *GAL80* inactivation events were readily detected in the midguts of old females and males, while very rarely present in young animals (Figures 1B and 1C). For further analysis, we focused on the clonal *GAL80* inactivation events having stem cell origin. Such somatic gene inactivation or LOH could occur via mitotic recombination, point mutation, gene deletion or epigenetic silencing. Recombination-based mechanisms of LOH such as crossing over, gene conversion (LaFave and Sekelsky, 2009), or break-induced replication (BIR) (Malkova and Ira, 2013) require a homologous chromosome. Therefore, we compared the frequency of *GAL80* inactivation events in females (two Xs) versus males (one X and one Y). Female midguts consistently had a higher frequency of at least one clonal *GAL80* inactivation event than male midguts. This increased over time from 12.7% in “young” (0 to 1 week; n = 158) to 68.5% in “aged” females (5 to 6 week; n = 181; Figure 1C). Multiple GFP-positive *GAL80* inactivation clones suggestive of several independent events were detected (Figures 1B and 1C). In contrast, males had no detectable clonal *GAL80* inactivation events in young flies (n = 114) and 2% in aged flies (n = 153; Figure 1C).

If the observed *GAL80* inactivation was due to homologous recombination-based mechanisms, LOH frequencies should dependent on the position of the marker gene on the chromosome arm: a distal gene would be more frequently exchanged during crossover events or copied via gene conversion or BIR. Consistent with that, the frequency of clonal GFP expression varied with the chromosomal location of the *GAL80*. 75% (n = 72) of aged females with *GAL80* at position 5B, 18 Mb from the centromere, had at least one clonal *GAL80* inactivation event, whereas only 40% (n = 75) did when *GAL80* was at position 19E, 2.5 Mb from the centromere (Table S1). Interestingly, the frequency of LOH at 19E is higher than predicted from meiotic recombination maps, which could suggest that additional factors influence *GAL80* inactivation somatically. Of note, *GAL80* position had no effect on the frequency of its inactivation in aged male midguts. Thus, a majority of the *GAL80* gene inactivation events did not occur in the absence of a homologous chromosome and varied with chromosomal location.

To explore further the possible mechanisms and in vivo impact of somatic mutations in ISCs, we exploited the phenotype of inactivation of Notch signaling components. Female flies heterozygous for a null allele of *Notch* (*N*^*55e11*^/+) present an overall wild-type midgut appearance. In contrast, midguts in which homozygous mutant *N*^*55e11*^/ *N*^*55e11*^ stem cells are genetically induced or express *N* RNAi (Figure S1) produce hyperplastic clusters of excess ISCs and enteroendocrine cells (EEs) that fail to properly differentiate (Micchelli and Perrimon, 2006, Ohlstein and Spradling, 2006). We therefore used the *N*^*55e11*^/+ genetic background to assess spontaneous LOH (Figures 1D–1I). As *N*^*55e11*^ is a recessive lethal allele and is located on the X chromosome, only female flies could be assessed. In young flies, only rare LOH events were observed (1.4% of midguts, n = 145; Figure 1G). In contrast, 80.9% of midguts of aged flies (n = 262) contained at least one LOH cluster (Figures 1E–1G). This was not observed in wild-type females (n = 519). Interestingly, the majority of guts (67.7%) had more than one event, with up to 25 clusters in a single gut detected (Figure 1F), similar to that observed for *GAL80* LOH (Figure 1C).

In order to test whether the LOH events were due to somatic homologous recombination, we then analyzed flies heterozygous for the *N*^*55e11*^ allele and a *GAL80* transgene inserted close to the *Notch* locus (position 5B) on the homologous chromosome (Figure 1H). We observed that in aged midguts 93.9% of LOH clones (n = 132) having the *Notch* phenotype were GFP positive, indicating concomitant inactivation of *GAL80* (Figure 1I). This suggests that the majority of LOH events arise due to a recombination event initiated along the chromosome arm between the centromere and the *GAL80* insertion site. The small fraction of LOH events (6.1%, n = 132) that displayed the *Notch* phenotype and were GFP negative could have arisen from recombination initiated within the 2-megabase region between the *Notch* locus and the *GAL80* or from recombination-independent gene-inactivating events.

LOH was not limited to the *Notch* gene or the X chromosome as it was also detected in flies heterozygous for other Notch pathway components like *neur* or *O-fut1* and *Delta* (Figure 1G and data not shown) on autosomes. As previously noted, in aged flies, 80.9% of *N*^*55e11*^/+ midguts had at least one LOH event, whereas 17.1% of *O-fut1*^*4R6*^/+ and 4.1% of *neur*^*IF65*^/+ midguts did (Figures 1G and 1J–1K; Table S1). In addition, a balancer chromosome, known to suppress recombination, reduced the frequency of LOH in *N*^*55e11*^/+ flies from 80.9% to 30.8% (*N*^*55e11*^/FM7, n = 120; Figure 1G). Once again, consistent with a strong contribution of recombination-based mechanisms, LOH frequencies varied with genomic position of the marker gene on the chromosome arm (Table S1). Although other differences such as the gene size of *Notch* relative to *O-fut1* and *neur* could influence LOH frequency, based on these data together with the *GAL80* results, we conclude that somatic homologous recombination based mechanisms like mitotic crossover or BIR lead to frequent LOH in ISCs. Interestingly, the rate near the centromere of the X chromosome was higher than that near the centromere of chromosome 3R, strongly suggesting that chromosomal differences also exist. Importantly, these analyses also revealed that additional gene inactivating events must occur, as spontaneous X-linked *GAL80* inactivation was also detected in males. We therefore decided to further explore the mechanisms underlying this type of event.

### Spontaneous Neoplasias Arise in Wild-Type Male Flies

**Figure 2:**
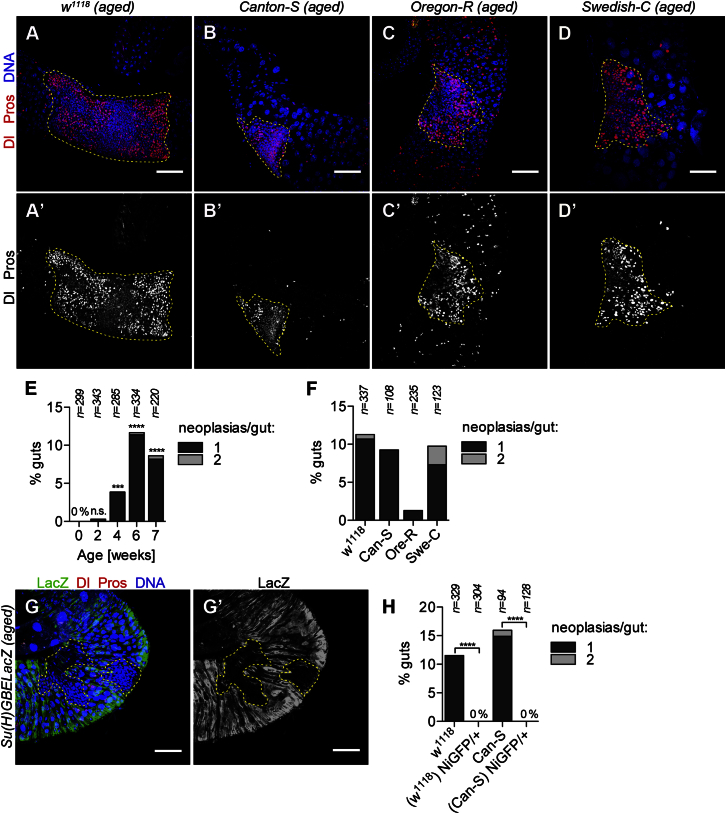
Inactivation of X-Linked *Notch* in Aged Male Midguts Leads to the Formation of Spontaneous Neoplasias (A–D′) Spontaneous clonal neoplasias in aged male midguts of the noted wild-type lines identified by Dl (cytoplasmic red in A, B, C, and D; white in A′, B′, C′, and D′) and Pros (nuclear red in A, B, C, and D; white in A′, B′, C′, and D′). (E) Frequency of ISC/EE neoplasias in *w*^*1118*^ male midguts at different ages. The 0 week time point was used to calculate statistical significance. (F) Frequency of ISC/EE neoplasias in aged males of wild-type strains at 5–6 weeks of age. (G and G′) Spontaneous male neoplasias did not activate the Notch reporter *Su(H)GBE-LacZ*. (H) Frequency of ISC/EE neoplasias in aged wild-type males of the indicated background with and without a *Notch* gene duplication (*NiGFP*). Neoplasias are outlined in yellow. Scale bars: 50 μm. ^∗∗∗^p < 0.001; ^∗∗∗∗^p < 0.0001; n.s., not significant (Fisher’s exact test, two-tailed). See also Figure S1.

In adult tissues, somatic inactivation of tumor suppressor genes poses a particular danger as it may lead to tumor formation. We reasoned that males might be at risk for inactivation of an X-linked tumor suppressor gene as only a “single hit” would be required. As we detected evidence for recombination-independent *GAL80* inactivation in males, we asked whether gene-inactivating events could lead to neoplasia in wild-type males. Consistent with this, we detected apparently clonal neoplasias composed of a cluster of ISCs and EEs in aged wild-type (*w*^*1118*^) males (Figures 2A and 2A′) but not in aged females (4 to 7 weeks, n = 290). The frequency of males with at least one neoplasia increased over time: 0% at eclosion (n = 299), 0.3% at 2 weeks (n = 343), 3.9% at 4 weeks (n = 285), 11.4% at 6 weeks (n = 334), and 8.2% (n = 220) at 7 weeks of age (Figure 2E). Neoplasias were highly variable in size ranging from 20 cells to several thousand cells comprising up to one-third of the intestine area and appeared identical in cellular phenotype to inactivation of X-linked *Notch*. At 6 and 7 weeks, rare midguts with two neoplasias were detected (Figure 2E). Neoplasias were also observed in males of *Canton-S*, *Oregon-R*, and *Swedish-C* wild-type lines with varying frequencies (Figures 2B–2D′ and 2F). In addition to the lower frequency of neoplasia detected in *Oregon-R*, we found that *w*^*1118*^ lines from different laboratory sources exhibited variable frequencies from approximately 5%–25%, suggesting that either subtle genetic differences or additional contributing factors influence neoplasia formation.

### Spontaneous Somatic Inactivation of *Notch* in Aging ISCs Leads to Male-Specific Neoplasia

We suspected that the spontaneous neoplasias were due to somatic inactivation of *Notch* since the cellular phenotype was reminiscent of inactivation of *Notch*; *Notch* is on the X chromosome; and neoplasias were detected in males but not females. Consistent with this, Notch pathway activation was not observed in the neoplasias (Figures 2G and 2G′, n = 14). Furthermore, addition of a transgene encoding a second genomic copy of *Notch* (*NiGFP*) on an autosome in males suppressed neoplasia formation in *w*^*1118*^ and *Canton-S* genetic backgrounds (Figure 2H), strongly implicating somatic inactivation of *Notch* in neoplasia formation. Consistent with previously published data (Bardin et al., 2010, Micchelli and Perrimon, 2006, Ohlstein and Spradling, 2006), the inactivation of *Notch* in stem cells but not enteroblast (EB) progenitor cells is sufficient to promote ISC/EE cell neoplasias (Figure S1). Therefore, the detected neoplasias probably arise through *Notch* inactivation in adult ISCs and not progenitor cells consistent with the notion that ISCs are the only frequently proliferating cell type in the adult intestine (Ohlstein and Spradling, 2006). There are an estimated 1,000 ISCs in the adult midgut and we detected at least one *Notch* inactivation event in ten guts suggesting that in males, somatic inactivation of *Notch* occurs at least once in every 10,000 ISCs and consequently leads to frequent formation of spontaneous neoplasias that perturb normal tissue architecture.

### The Frequency of Male-Specific Neoplasias Correlates with ISC Proliferation Rates

**Figure 3:**
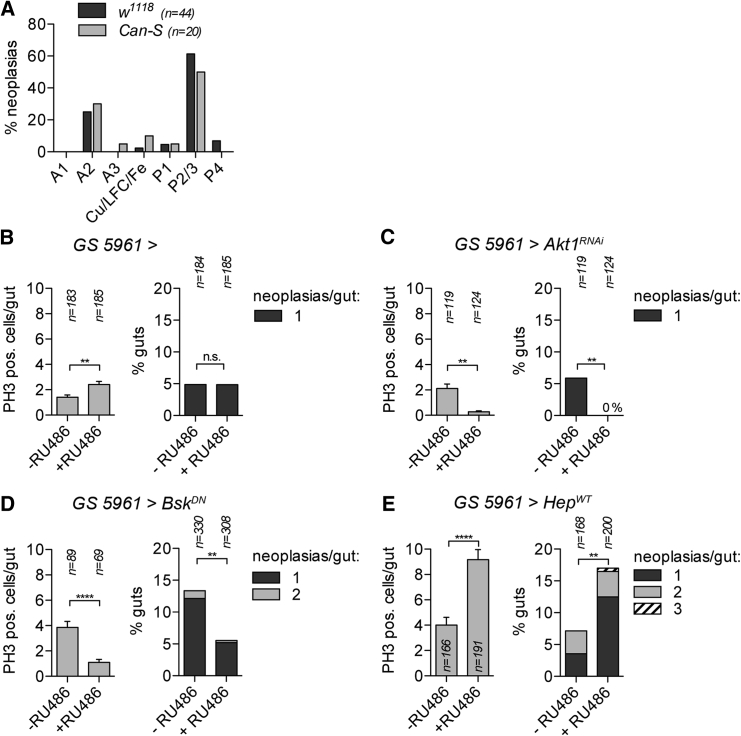
Frequencies of Spontaneous Male *Notch* Inactivation Correlate with ISC Proliferation Rates (A) Distribution of neoplasias in midgut regions in *w*^*1118*^ and *Canton-S* males. n = total number of neoplasias analyzed. (B–E) Frequencies of neoplasias and proliferation rates (phospho-Histone3-positive cells per gut) were quantified in 5- to 6-week-old males aged on control food (−RU486) or food inducing GAL4 expression in stem and progenitor cells (+RU486). RU486 had no effect on neoplasia formation in control flies (B). RU486-induced expression of Akt1^RNAi^ (C) or Bsk^DN^ (D) decreased ISC proliferation rates and frequencies of neoplasia. Expression of Hep^WT^ (E) increased proliferation rates and neoplasia frequency. Error bars indicate SEM, ^∗∗^p < 0.01, ^∗∗∗∗^p < 0.0001; n.s., not significant (t test, two-tailed for PH3; Fisher’s exact test, two-tailed for neoplasia frequency). See also Figure S2.

Errors arising during ISC division could lead to the detected mutations, and thus proliferation rate would influence the frequency of mutation. Stem cell proliferation rates have been previously demonstrated to vary along the anterior-posterior axis, with the highest rates in the anterior region 2 (A2) and the posterior regions 2 and 3 (P2/3) (Marianes and Spradling, 2013). Consistent with ISC proliferation rate influencing neoplasia formation, neoplasias in *w*^*1118*^ and *Canton-S* were detected more frequently in the A2 and P2/3 regions (Figure 3A).

In order to further investigate the relationship between ISC proliferation and neoplasia formation, we then tested the effects of *Ecc15*, paraquat, and bleomycin. Surprisingly, with our treatment conditions neither *Ecc15* nor paraquat induced a strong proliferative effect in adult male intestines, in contrast to what has been reported for adult female intestines (Amcheslavsky et al., 2009, Buchon et al., 2009b), and neither affected the frequency of neoplasias (Figure S2). Bleomycin feeding altered proliferative response and had a slight impact on neoplasia frequency; however, this was not statistically significant (Figure S2). It is possible that these treatments had a minimal impact on the total number of ISC divisions because punctual exposures could only be used to minimize overall toxicity and avoid lifespan reduction.

We therefore tested the effect of continuous modulation of stress signaling in the gut with genetic means. To do so, we used the 5961 Gal4 GeneSwitch system in which ISC and EB progenitors express Gal4 whose activity is controlled by addition of RU486 (Mathur et al., 2010, Osterwalder et al., 2001), allowing the comparison of flies with identical genetic backgrounds. In control flies, RU486 feeding did not affect neoplasia formation though we observed a mild increase in proliferation at a late 5-week time point (Figure 3B). To test the effect of decreasing proliferation, we inhibited the Insulin pathway using *Akt1* RNAi, (Biteau et al., 2010). We found that neoplasia frequency went from 5.9% in uninduced flies (n = 119) to 0% in *Akt1* RNAi induced flies (n = 124), coincident with decreased proliferation (Figure 3C). Similarly, overexpression of a dominant-negative form of *basket*, which decreases Jnk signaling and slows down stem cell proliferation rates (Biteau et al., 2010), decreased the percentage of male midguts with neoplasia from 13.3% in controls (n = 330) to 5.5% in induced flies (n = 308, Figure 3D). To test the effects of increased ISC proliferation, the Jnk signaling pathway was activated through overexpression of *hep* (Biteau et al., 2010). The frequency of neoplasia increased from 7.1% in control males (n = 168) to 17% in RU486-fed males (n = 200; Figure 3E).

Taken together, our data suggest that neoplasia formation correlates with gut regions having higher rates of ISC proliferation and is affected by modulation of Insulin and Jnk signaling pathways, correlating with stem cell proliferation rates.

### Genomic Deletions and Large Structural Rearrangements Underlie Loss of *Notch* in Aging Male Stem Cells

**Figure 4:**
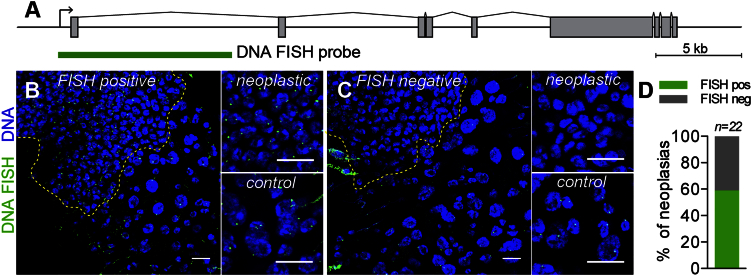
DNA FISH Reveals Genomic Disruption of *Notch* Locus in a Subset of Neoplasias (A) The *Notch* locus and the 10 kb DNA FISH probe in green. (B–D) DNA FISH was performed on aged male midguts with neoplasia (detected by accumulation of *Prospero*^*V1*^*Gal4 > GFP*-positive cells). Higher-magnification views of neoplastic and control regions of the same tissue. 13 out of 22 neoplastic midguts were DNA FISH positive (B) and 9 DNA FISH negative (C). (D) Quantification. Yellow lines indicate borders between neoplastic and surrounding tissue. Scale bars: 10 μm.

We next sought to determine the molecular nature of the male-specific genomic aberrations of the *Notch* locus by both DNA fluorescence in situ hybridization (FISH) and deep sequencing approaches. Using a DNA FISH probe corresponding to the first 10 kb of *Notch* (Figure 4A), we found that 9 out of 22 neoplasias lacked a DNA FISH signal in neoplastic tissue, but not in adjacent tissue, pointing to the possibility of genomic deletions of the *Notch* region in these neoplasias (Figures 4B–4D).

**Figure 5:**
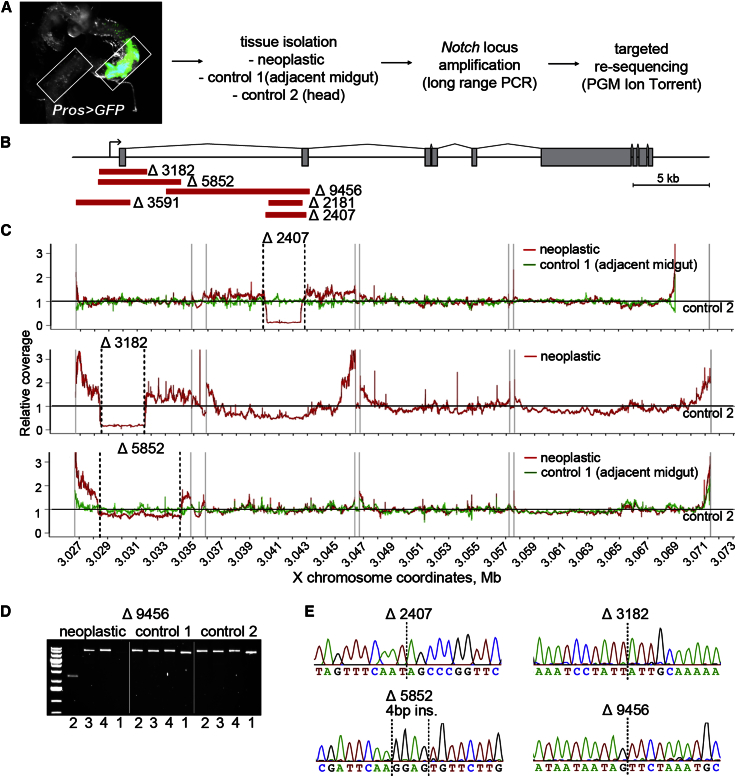
Genomic Deletions Underlie Loss of *Notch* Activity in Spontaneous Male Neoplasias (A) *Prospero*^*V1*^*Gal4 > GFP* positive cells identified neoplasias with samples containing 40%–80% of neoplastic cells. Adjacent midgut (control 1) or head (control 2) tissues were also isolated. Deep sequencing was performed on PCR-amplified DNA spanning the *Notch* locus. (B) The *Notch* genomic region. Red lines depict 6 deletions identified in male neoplasias. (C) Coverage at each position obtained for the head control sample (control 2) was set as reference (black line set to 1) and relative coverage for neoplastic (red) and adjacent midgut (green) were plotted. Coverage plots revealed a decreased number of mapped reads over the region of deletions (Δ2407, Δ3182, and Δ5852) in three neoplastic samples. The adjacent midgut control was not available for the sample with Δ3182 deletion. Black lines indicate deletion break points and gray lines represent the boundaries of PCR amplicons. (D) Agarose gel of four PCR amplicons spanning the *Notch* locus amplified on neoplastic and control tissue DNA. The Δ9456 deletion in the neoplastic cells removed the reverse primer site of the first amplicon (no amplification) and produced a shortened second amplicon. DNA ladder range: 20,000-700 bp (with 5,000 kb and 1,500 kb bands of higher intensity). (E) Sanger sequencing confirming the breakpoints of deletions shown in (C) and (D). Black lines indicate deletion break points. The Δ5852 deletion had an insertion of GGAG sequence at the breakpoint. See also Figure S3.

We then isolated DNA of 12 flies from microdissected tissue enriched for neoplastic cells using adjacent midgut tissue and the head as control samples (Figure 5A). The 45 kb *Notch* locus was amplified as four long-range PCR products and deep sequenced to 1,200× using Ion Torrent technology. This revealed deletions of 2.4, 3.2, and 5.9 kb in three of the neoplastic samples detected as a drop in read coverage in the neoplastic sample (Figures 5B and 5C). In the remaining samples sequenced, no SNVs or small INDELs that could explain *Notch* inactivation were detected. PCR amplification of 18 additional samples detected another three deletions of 2.2, 3.6, and 9.5 kb specific for neoplastic cells (Figures 5B and 5D; Figure S3). The breakpoints of all six identified deletions were confirmed by Sanger sequencing (Figure 5E; Figure S3). Our lack of detection of mutations in the remaining analyzed samples could have resulted from failure of amplification of mutant DNA due to larger or more complex genomic structural variants.

**Figure 6:**
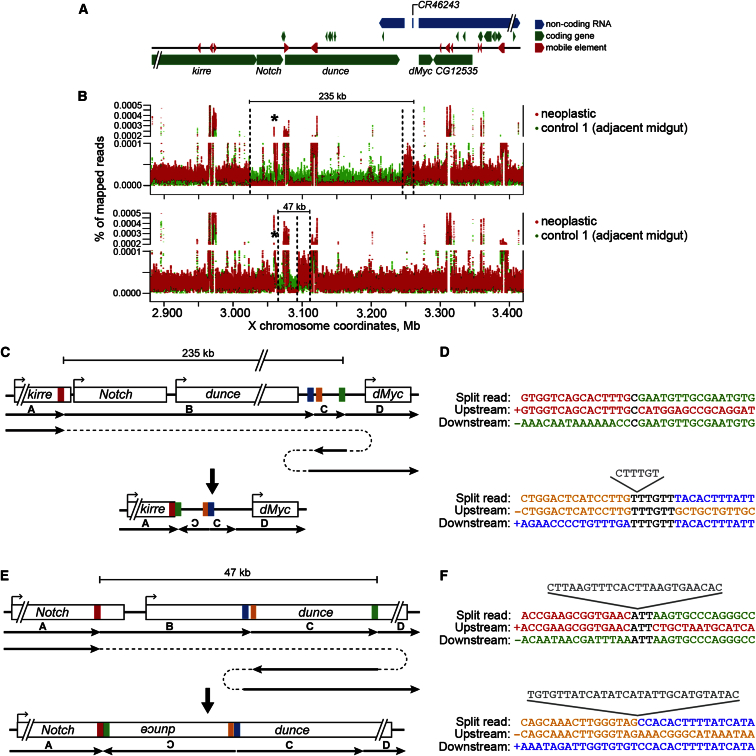
Whole-Genome Sequencing of Male Neoplasias Reveals Spontaneous Complex Genomic Rearrangements Encompassing the *Notch* Locus (A) The surrounding genomic region of the *Notch* locus. Coding genes, green; non-coding RNAs, blue; mobile transposable elements, red. (B) Sequencing coverage for the genomic region in (A) was plotted for neoplastic (red) and adjacent midgut control (green) samples. Dashed lines represent borders between identified regions of coverage drops (deletions) or increases (duplications) in the neoplastic samples. Asterisks indicate the 5 kb region of unknown nature seemingly amplified in neoplastic cells. Note: other peaks throughout the plots correspond to multiply mapped sequences of transposable elements and are present in all samples. (C–F) Two complex genomic rearrangements identified in neoplastic cells. Schemes (C) and (E) represent genomic regions before and after the rearrangement. Arrows and dashed lines indicate the order and direction of sequences. Sequence regions participating in identified breakpoints are indicated with red, green, orange, and blue bars. In both cases, a deletion of a central region B (containing a part or entire *Notch* locus), could be explained through a template-switching event that duplicates the region C in an inverted orientation, followed by a second template-switch linking the inverted C region to C in the correct orientation. (D and F) Nucleotide sequences of breakpoint junctions in split-reads and corresponding wild-type upstream and downstream genomic sequences participating in the rearrangements. Colors correspond to the genomic regions in (C) and (E). Breakpoint microhomologies are in black, flanking microhomologies are underlined, and sequences of breakpoint insertions are in gray above the corresponding breakpoints. See also Figure S4.

To assess the role of additional types of genomic alterations, we performed whole-genome paired-end Illumina sequencing on three additional neoplasias, each with adjacent tissue and head controls. Copy number variation throughout the genome was assessed using Control-FREEC (Boeva et al., 2012, Boeva et al., 2011). Potential regions of genomic loss were further analyzed. After exclusion of false positives linked to multiply mapped transposable element reads, the only large genomic sequence losses that could be detected spanned part or all of the *Notch* locus on the X chromosome in all three samples (Figures 6A and 6B, data not shown), suggesting the possibility of large-scale gene deletion events spanning 40–500 kb. Large regions with reduced read coverage suggestive of genomic deletion were apparent in neoplastic samples but not in adjacent tissue or head controls (Figures 6A and 6B; Figure S4), coincident with those detected by Control-FREEC. One sample was not analyzed further due to lack of informative reads at apparent breakpoint junctions (data not shown).

The first neoplasia had an apparent deletion of greater than 200 kb, which arose in the *kirre* gene adjacent to *Notch* and spanned the *Notch* and *dunce* loci. We then examine aberrant reads and split-reads within the vicinity of the apparent deletion junctions. This analysis supported a genomic structure resulting in loss of the *Notch* and *dunce* genes, as well as additional genes present at these genomic regions, and a duplication of non-coding RNA *CR46243* adjacent to *Myc* (Figures 6A and 6C). In addition, there was an apparent amplified region of about 5 kb within the large exon of the *Notch* gene (Figure 6B) that was likely not present at this genomic location since the reads at flanking locations showed no evidence of joining the adjacent segments of the annotated genome. Unfortunately, we could not determine whether this segment was localized elsewhere in the genome or present extrachromosomally as no informative reads were found at the junctions.

Analysis of second neoplastic sample revealed a similar type of structural rearrangement. Aberrant and split reads showed evidence for a fusion of the coding region of *Notch* within the sixth exon to a 17 kb inverted duplicate portion of the *dunce* locus 47 kb away, which thereby removed 30 kb of genomic sequence including the 3′ portion of the *Notch* gene and adjacent genes. Additional read sequences further supported the fusion of this duplicated region to a sequence 1.4 kb away in *dunce*, in the normal orientation (Figures 6A and 6E). Interestingly, it appears as if the same 5 kb portion of *Notch* was amplified in this neoplastic sample, though the exact structure of this in the genome could not be determined with certainty.

Altogether, these data reveal that *Notch* becomes somatically inactivated in ISCs by both small deletions (2–10 kb) and genome rearrangements leading to large-scale deletions (50–200 kb) and inversions. Further studies will be needed to determine quantitatively the relative contribution of each type of deletion to neoplasia formation in vivo.

### Molecular Mechanisms of Somatic X-Linked *Notch* Deletions

We next considered the origin of the somatic deletions in *Notch*. The simple deletions identified (Figure 5) could be produced by excision of a transposon. However, we found no evidence of a transposon in this region, though de novo insertion and deletion cannot be excluded. Homologous recombination mechanisms can be ruled out as breakpoint junctions lacked extended homology. Instead, in 5 out of 6 deletions breakpoints junctions or flanking DNA had microhomology sequences (Figure S3D). This argues against the use of the classic non-homologous end-joining pathway, which typically results in fusions lacking homology. Additionally, 2 out 6 breakpoints of simple deletions and 1 complex rearrangement had short inserted sequences, possibly locally templated (Figure S3D; Figures 6D and 6F). Microhomology sequences and short inserts are typical of an alternative NHEJ pathway or a pathway involving erroneous DNA replication: “fork stalling and template switching” (FoSTeS) (Lee et al., 2007) or “microhomology mediated break-induced replication” (MMBIR) (Hastings et al., 2009, Payen et al., 2008). Importantly, the two complex rearrangements (Figure 6) have deletions coupled to inverted duplications that can be best explained by the template switching of a replicative polymerase along the DNA (FoSTeS/MMBIR) (Lee et al., 2007) and would be difficult to explain by an alternative NHEJ model. Interestingly, the complex rearrangements in the *Notch* locus detected here, albeit more simple in nature, show similarities to complex rearrangements detected in humans (Conrad et al., 2010, Lee et al., 2007) and to chromothripsis occurring in cancers whereby a single or a few chromosomes are affected by locally clustered deletions, inversions, and duplications with breakpoints sharing microhomology (Holland and Cleveland, 2012, Kloosterman et al., 2011b, Stephens et al., 2011, Yang et al., 2013).

## Discussion

Our study provides insight into the frequency and causes of spontaneous genetic instability in adult stem cells and its contribution to the onset of neoplasia. We reveal surprisingly frequent genetic instability in vivo in adult *Drosophila* ISCs through at least two mechanisms. First, LOH mediated by a mitotic homologous recombination-based mechanism arises very frequently, occurring in 80% of flies for a distal gene on the X chromosome. Such a high frequency of recombination-based LOH suggests that in ISCs DNA damage occurs along the chromosome arm and is frequently repaired with use of the homologous chromosome through recombination-based mechanisms such as cross-over or BIR. A high frequency of DNA breaks is consistent with a previous report of increased levels of γH2Av detected during midgut aging (Park et al., 2012). Second, homologous recombination-independent gene inactivation in ISCs is due at least in part to deletion events, leading to spontaneous inactivation of *Notch* and driving neoplasias in 10% of aged wild-type males. Gene deletion events can also be produced in the context of chromosomal rearrangements leading to deletion, inversion, and duplication of the genomic region surrounding the *Notch* locus. Significantly, our findings differ markedly from previous reports of “tumors” in aged male and female fly midguts, consisting of a thickened tissue (Garcia et al., 2011), and from midgut dysplasia during aging caused by stimulation of ISC proliferation due to gut microbiota (Biteau et al., 2008, Buchon et al., 2009a). The neoplasias that we identified occur only in males, consist of ISC/EE cells, and have a clear somatic genetic origin. Importantly, we find that gene-inactivating events are not limited to the *Notch* locus, as inactivation of *GAL80* in males also occurs. Thus, it appears highly likely that somatic mutation of many other genes may also alter midgut physiology and homeostasis during aging.

Growing evidence suggests important effects of somatically acquired mutations on adult stem cells, tissue renewal, and tissue function during aging. DNA double-strand break accumulation accompanied by replication stress has been clearly demonstrated in aging hematopoietic stem cells (Beerman et al., 2014, Flach et al., 2014, Rossi et al., 2007, Rübe et al., 2011), though whether frequent somatic mutation results is not yet clear. Similarly, in telomerase-compromised animals, telomere attrition leads to activation of DNA damage checkpoints resulting in defective adult stem cell function and tissue renewal, which likely contributes to functional stem cell decline during aging (Fumagalli et al., 2012, Lee et al., 1998, Rudolph et al., 1999, Sperka et al., 2012). Additional evidence exists that mutations, presumably affecting stem or progenitor cells, can lead to selection of lineages in the hematopoietic compartment, male germline, and skin (Busque et al., 2012, Genovese et al., 2014, Goriely et al., 2003, Holstege et al., 2014, Hsieh et al., 2013, Jacobs et al., 2012, Jaiswal et al., 2014, Jan et al., 2012, Laurie et al., 2012, Martincorena et al., 2015, Welch et al., 2012) and reviewed in (Adams et al., 2015), implying an increased relative fitness of these mutant lineages. Our work demonstrates that, in *Drosophila* intestinal stem cells, frequent inactivation of *Notch* leads to a significant growth advantage of stem cell descendants over their wild-type counterparts. An obvious corollary to these findings is the notion that mutations leading to suboptimal growth may cause stem cell functional decline or loss from the tissue through cell competition mechanisms, consistent with the long-standing DNA damage theory of aging.

During aging in flies, ISC proliferation is increased (Biteau et al., 2008). Our data suggest that increased ISC proliferation can lead to an increased frequency of spontaneous mutation. The number of stem cell divisions has recently been shown to correlate well with the lifetime cancer risk (Tomasetti and Vogelstein, 2015). Our data raise the possibility that “healthy” renewing mammalian somatic tissues, apart from accumulating point mutations, may also be frequently affected by mitotic recombination as well as genomic deletions, which would not be easily detected in exome sequencing studies routinely performed on the mammalian genome but is supported by data using SNP analysis (Hsieh et al., 2013, Jacobs et al., 2012, Laurie et al., 2012, Martincorena et al., 2015, O’Huallachain et al., 2012).

Intriguingly, we have found that apart from simple deletions, complex chromosomal rearrangements also contribute to somatic *Notch* inactivation in aging ISCs. These rearrangements, though apparently simpler in nature, have shared features with chromothripsis recently shown to occur in cancers and congenital disorders (Liu et al., 2011, Stephens et al., 2011), including clustered breakpoints with deletions, inversions, and duplications having both microhomology and small inserted sequences at breakpoint junctions. While the rearrangements detected here are less complex compared to the hundreds of rearrangements sometimes detected in chromothripsis, could similar underlying mechanisms be involved in their generation? Mechanistically, genomic rearrangements driven by microhomology-mediated pathways might preferentially affect lone chromosomes lacking a homolog that could be used for homologous recombination-based repair such as when a chromosome is isolated in a micronucleus following defective mitosis, recently proposed to drive chromothripsis (Stephens et al., 2011, Zhang et al., 2015). Similar lone chromosomes are present during meiosis, in haploid yeast, and in case of the X chromosome of male flies studied here, during which similar complex rearrangements have been found to occur (Kloosterman et al., 2011a, Payen et al., 2008). In these instances, a collapsed replication fork or single broken DNA end might be repaired through employment of the MMBIR machinery, a strategy that may satisfy the DNA damage checkpoint, but ultimately lead to gross chromosomal rearrangement.

Altogether, our findings of surprisingly high rates of somatic mutation have important implications for in vivo somatic cell genome stability, suggesting that genetic mosaicism may be more prevalent and have a greater impact on adult tissues during the aging process than previously suspected. The adult fly intestine provides a useful model system for aging studies in order to address these questions.

## Experimental Procedures

### *Drosophila* Stocks and Aging

Fly stocks used in this study can be found in the Supplemental Experimental Procedures.

Flies were maintained at 25°C on a standard medium. For aging experiments, flies were crossed in standard vials (10–15 females/vial) and newly eclosed progeny were collected over 2–4 days. Flies (mixed males and females of equal starting number) were then aged in plastic cages (10 cm diameter, 942 ml, 700–900 flies/cage). Freshly yeasted food was provided in petri dishes every 2–3 days. Every 7 days, flies were transferred to clean cages. Dead flies were scored upon each food change to assess survival rates. If not otherwise mentioned, young flies were assayed at 4–7 days of age (0–1 week) and aged flies were 35–40 days old (5–6 weeks).

For GeneSwitch experiments (Figure 3), crosses were raised in a standard food and newly eclosed siblings were sorted and shifted on food supplemented with 50 μg/ml of RU486 in EtOH or EtOH alone.

### Immunofluorescence and Quantifications

Midgut fixation and immunofluorescence staining were performed as described previously (Bardin et al., 2010). *GAL80* inactivation events occurring in stem cells were scored as clusters of at least four GFP-positive cells. Of note, single ECs showing *GAL80* inactivation were detected but not scored as they did not occur in stem cells. LOH events in females heterozygous for *N, neur,* or *O-fut1* as well as male spontaneous neoplasias were scored as clusters of at least 20 Delta and/or Prospero positive diploid cells. Wild-type females were observed at all time points and neoplasias were not detected (n = 519 females). Quantifications were performed on entire midguts.

### Male Neoplasia Isolation and Sequencing

*Pros*^*V1*^*Gal4 UAS-2XGFP* males were used to visually identify midguts containing neoplasias. The midgut region containing an estimated 40%–80% neoplastic cells was manually dissected together with the neighboring control gut tissue as well as the fly head. Genomic DNA was isolated and amplified for targeted *Notch* or whole-genome sequencing as specified in the Supplemental Material.

## Author Contributions

K.S. and A.J.B. designed the study and analyzed all experiments. A majority of experiments were done by K.S. A.J.B. helped with quantification and genome sequencing analysis. S.N. conducted initial DNA FISH experiments and provided data for Figures 1E–1G, 1J, 2E–2G, and Table S1. L.G. contributed to Figures 3, S1, and S2. P.S. made important transgenic lines and provided data for Figure S3A–S3C and S4B–S4D. D.S. contributed data for Figures 1C and 3C. M.Z. provided data for Figure 2F. S.L., V.B., and T.R.F. provided suggestions for IonTorrent sequencing. V.B. made the plots in Figure 5C. S.L. made the plots of mapped reads in Figures 6B and S4A. A.J.B. and K.S. wrote the manuscript.

## Accession Numbers

The accession number for the whole-genome sequencing of male neoplasias reported in this paper is ArrayExpress: E-MTAB-3917.

## Supplemental Information

Document S1. Supplemental Experimental Procedures, Figures S1–S4, and Table S1

Document S2. Article plus Supplemental Information
