# Supplementary material for: Frequent Somatic Mutation in Adult Intestinal Stem Cells Drives Neoplasia and Genetic Mosaicism during Aging
Source: Cell Stem Cell. 2015 Dec 3;17(6):663–74. doi: 10.1016/j.stem.2015.09.016 (PMC5138153; doi:10.1016/j.stem.2015.09.016)
Supplement: Document S1. Supplemental Experimental Procedures, Figures S1–S4, and Table S1 [file mmc1.pdf]

Cell Stem Cell
Supplemental Information

Frequent Somatic Mutation in Adult Intestinal Stem Cells Drives Neoplasia and Genetic Mosaicism during Aging

Katarzyna Siudeja, Sonya Nassari, Louis Gervais, Patricia Skorski, Sonia Lameiras, Donato Stolfa, Maria Zande, Virginie Bernard, Thomas Rio Frio, and Allison J. Bardin

Supplemental Data

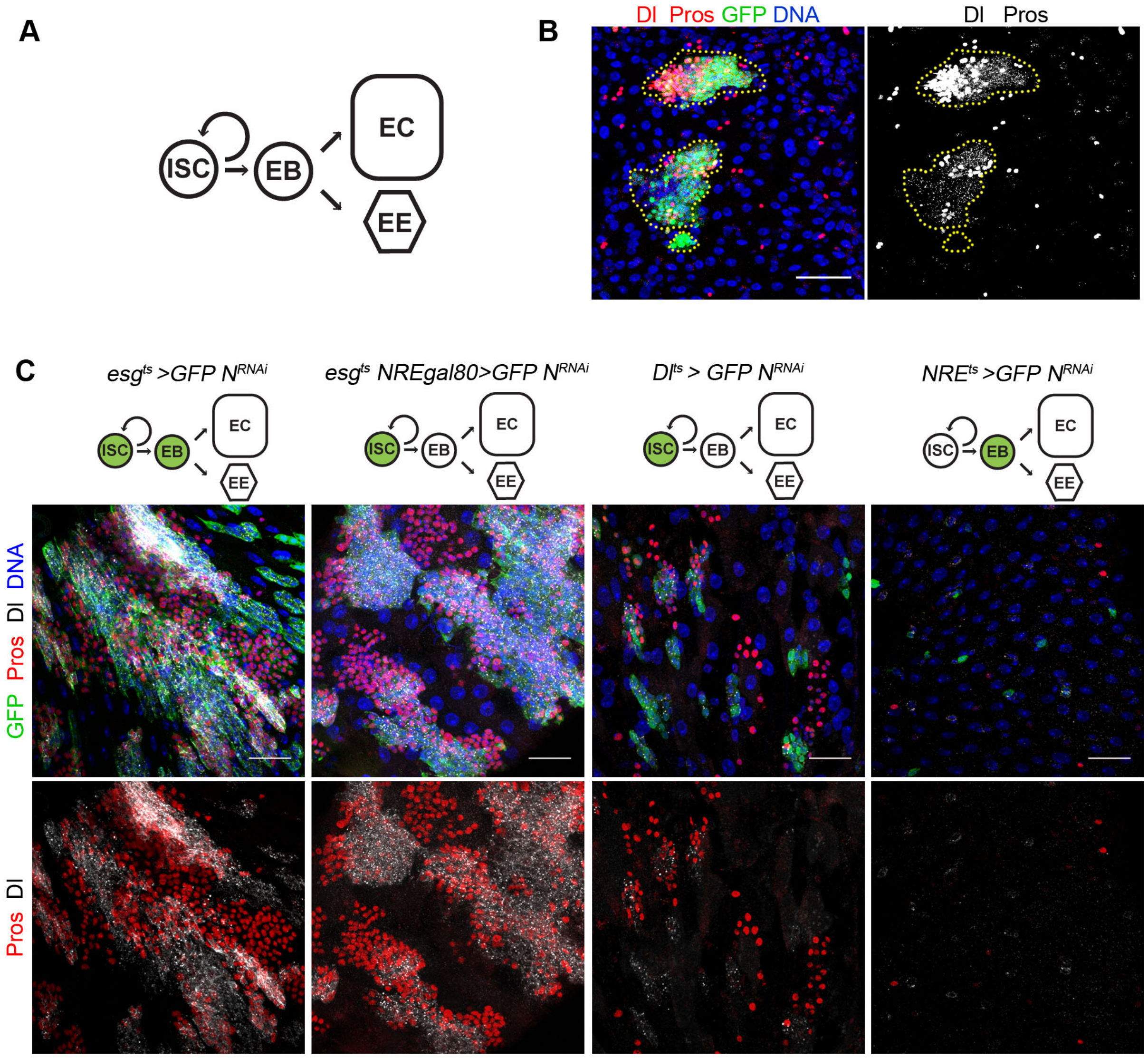

Figure S1. *Notch* inactivation in ISCs leads to ISC/EE neoplasias. Related to Figure 1 and 2.

(A) Simplified scheme of *Drosophila* intestinal stem cell (ISC) lineage. The ISC divides to self renew and generates an enteroblast (EB), a postmitotic progenitor, which further differentiates into one of the two differentiated cell types: an enterocyte (EC) or an enteroendocrine cell (EE). (B) Clonal RNAi knockdown of *Notch* in ISCs marked by GFP (green, outlined in yellow) leads to increased numbers of Dl+ ISCs (cytoplasmic red) and Pros+ EE cells (nuclear red) and loss of enterocytes (large nuclei) as previously demonstrated (Micchelli and Perrimon, 2006; Ohlstein and Spradling, 2006). (C) Expression of *Notch* RNAi in ISCs and EBs (*esgGAL4, tubGAL80ts*) or ISCs alone (*esgGAL4, tubGAL80ts, NRE-GAL80* and *DlGAL4, tubGAL80ts*) but not in EBs (*NRE-GAL4 tubGAL80ts*) leads to ISC/EE neoplasias. *Notch* RNAi together with GFP was induced in adult flies for 12 days using combinations of cell type specific temperature sensitive *GAL4* drivers and *GAL80* repressors. Scale bars: 25 μm.

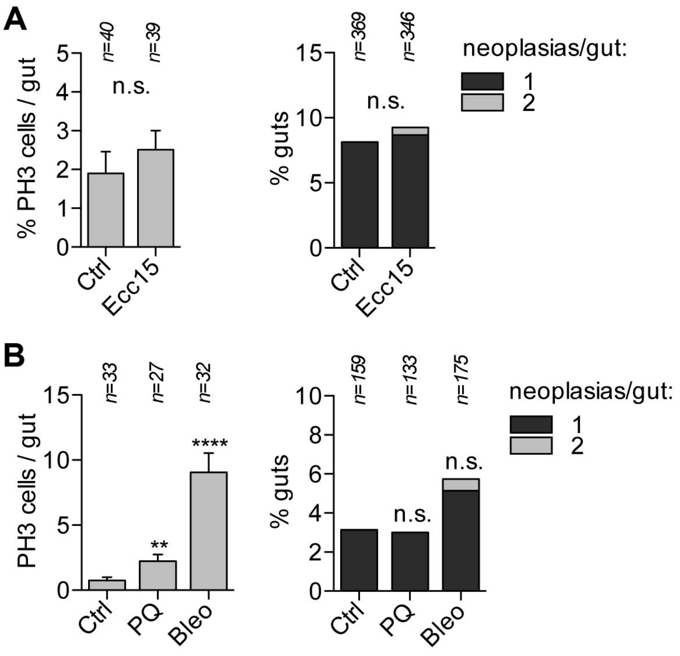

Figure S2. Treatment with *Ecc15*, paraquat and bleomycin do not impact the frequencies of spontaneous neoplasias in aged wild-type males. Related to Figure 3.

Adult flies were treated with *Ecc15* in (A), or paraquat or bleomycin in (B) or sucrose alone as a control. Proliferation was assayed by phospho-histone 3 (PH3) staining in young 1-2-week-old flies after 48 hours of treatment. For the quantification of neoplasias flies were aged for 5 weeks with two 48-hour-treatments each week until week 4, followed by a week of recovery.

Canton-S males were used in all the experiments. Error bars represent SEM. ** $p<0.01$; **** $p<0.0001$, n.s.- not significant (t-test, two-tailed for PH3 and Fisher's exact test, two-tailed for neoplasia frequency).

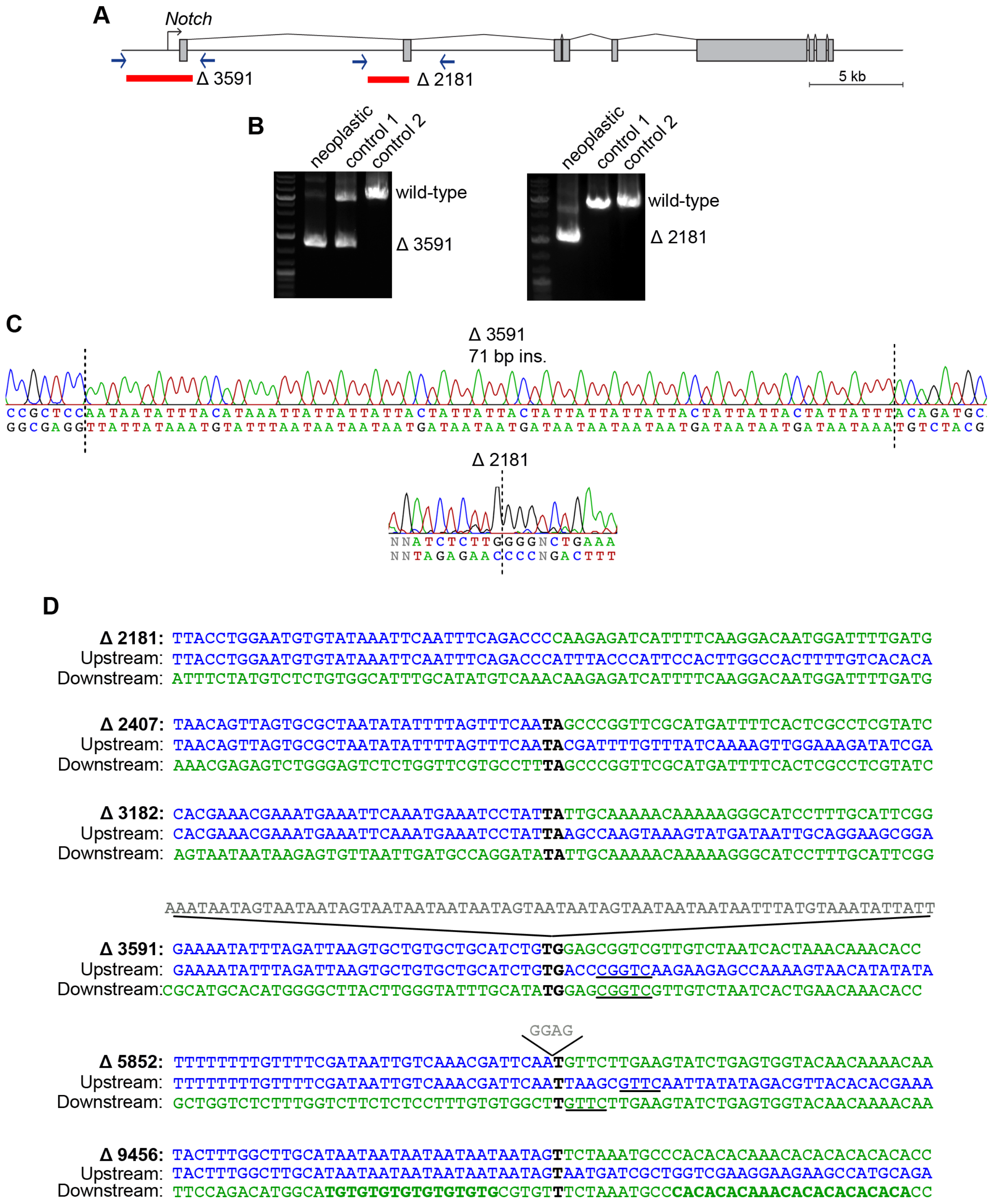

Figure S3: Validation of breakpoint sequences of neoplastic deletions identified by targeted *Notch* sequencing. Related to Figure 5.

(A) Schematic representation of the *Notch* locus and two deletions (Δ3591 and Δ2181, in red) identified in male neoplasias. Blue arrows indicate primer sites used to PCR-amplify wild-type and mutant genomic fragments on the original genomic DNA samples. (B) Agarose gels verifying the presence of deletions in neoplastic samples. Control 1 is adjacent midgut DNA and control 2 is head

DNA. For Δ3591 deletion contamination of adjacent midgut control with some neoplastic cells is apparent. Primer pairs used were:
GAGCACTAAGAATGTGACTGCTTTCGTTTGT/ ACTCGAGCTCAGGAAATGCC (for Δ3591);
AGTGATCTTTACCTGGAATGTG/ GAATCGCTCTCGTTGTTGGC (for Δ2181).
(C) Sanger sequencing of identified deletion breakpoints. Black dashed lines indicate deletion break points. The Δ5391 deletion was accompanied by the insertion of a 71bp sequence at the breakpoint.
(D) The breakpoint sequences of six somatic deletions are shown. For each breakpoint sequence, upstream genomic sequences flanking the deletion site are shown in blue, and downstream sequences are in green. Breakpoint microhomologies are in black, flanking microhomologies are underlined and sequences of breakpoint insertions are in grey above the corresponding breakpoints. The inverted repeat structure found around the downstream breakpoint of the 9456bp deletion is indicated with bold letters. Breakpoint microhomologies make it impossible to define exact break points as microhomology sequence can be derived from either upstream or downstream templates.

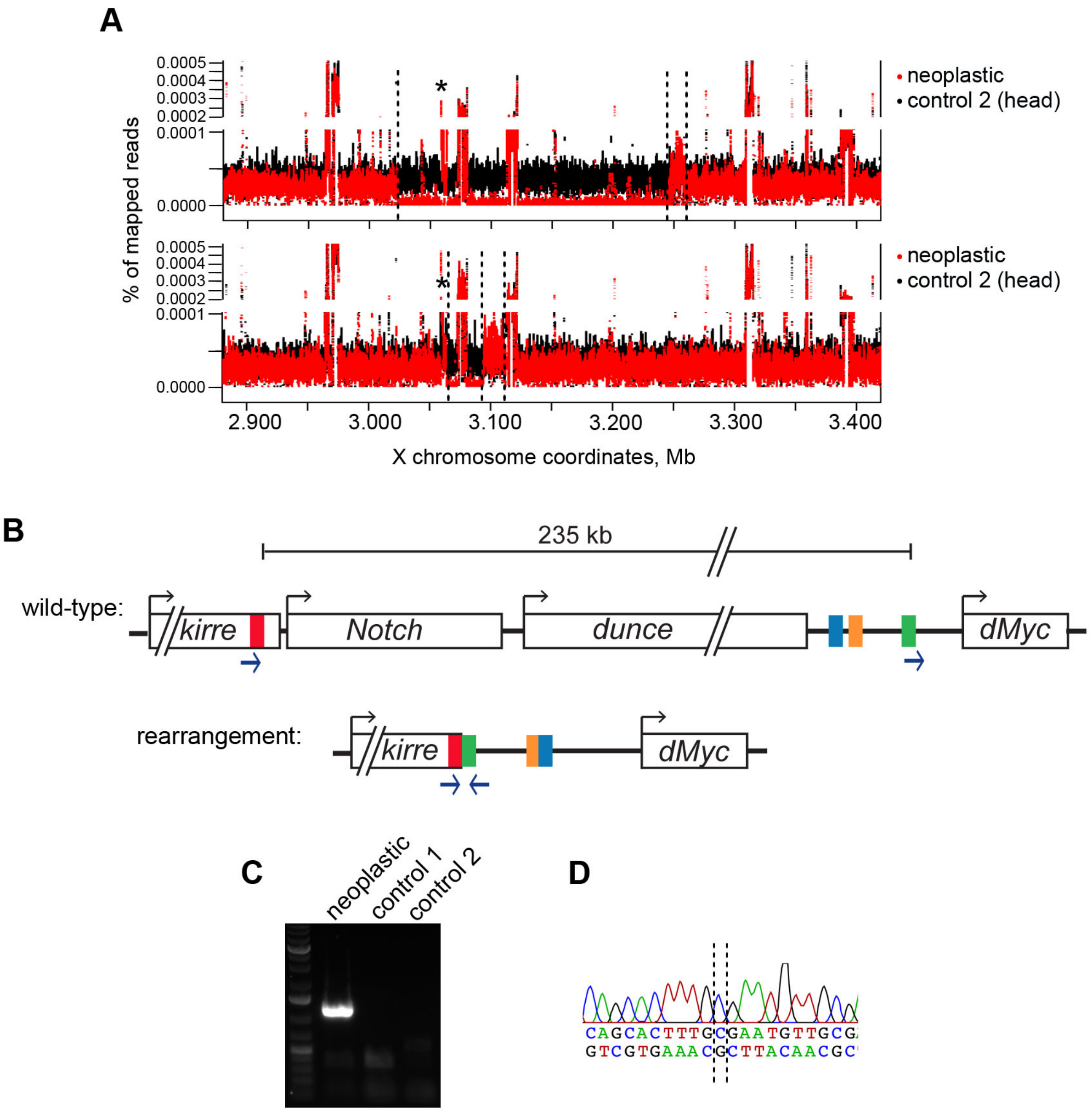

Figure S4: Coverage plots for neoplastic rearrangements and head DNA controls and PCR validation of an inverted junction identified by whole-genome sequencing. Related to Figure 6.

(A) Sequencing coverage for the genomic region containing identified rearrangements was plotted for neoplastic (red) and head control (black) samples. Dashed lines represent borders between identified regions of coverage drops (deletions) or increases (duplications) in the neoplastic samples. Asterisks indicate the amplification of a 5kb region within the 6th exon of *Notch* in neoplastic cells (B) Schematic representation of the genomic region of interest before and after the rearrangement. Blue arrows indicate primer sites used to PCR-amplify wild-type and mutant genomic fragments on the original genomic DNA samples. (C) Agarose gels verifying the presence of an inverted junction in the neoplastic sample. Control 1 is adjacent midgut DNA and control 2 is head DNA. (D) Sanger sequencing of identified deletion breakpoints. Black dashed lines indicate potential break points (the exact break point can not be identified due to a presence of a 1-base breakpoint microhomology).

LOH marker	Chromosome	Cytological location	Approx. distance from the centromere	% midguts with LOH clones					n*
				1 clone	2-3 clones	4-5 clones	>5 clones	≥1 clone	
GAL80 transgene									
tubGAL80	X	1E	22 Mb	27.1	27.1	10.5	3.9	68.5	181
tubGAL80	X	5B	18 Mb	22.2	38.9	9.7	4.2	75.0	72
tubGAL80	X	19E	2.5 Mb	24	12.0	0	4.0	40.0	75
Notch pathway components									
N	X	3C	20 Mb	13.4	27.9	24.4	15.3	80.9	262
O-fut1	2R	50E	14 Mb	13.6	3.4	0	0	17.1	88
neur	3R	85C	9 Mb	4.1	0	0	0	4.1	245

Table S1. Frequency of LOH events for different markers used in this study. Related to Figure 1.

LOH clonal events (GFP positive for *GAL80* LOH or showing Notch loss of function phenotype for *N*, *O-fut1* and *neur*) were scored in female midguts at 5-6 weeks of age. *n = number of midguts analyzed

Supplemental Experimental Procedures

Drosophila stocks

The following fly stocks and alleles were used in this study. From the Bloomington stock center: w^{1118}; *Canton-S*; *Oregon-R*; *Swedish-C*; *UAS-2XGFP; UAS-Akt1 RNAi; UAS-BskDN, UAS-N RNAi*. The following stocks were generous gifts: N^{55e11}, $neur^{IF65}$, a *Notch* rescue BAC on chromosome 2 *[NiGFP]* (Couturier et al., 2012) (F. Schweisguth); *tubGal80, actGal4 UAS-GFP* (R. Xi) (the *tubGal80* inserted at position 1E (Lee and Luo, 1999)) *Su(H)GBE-lacZ* (S. Bray); $Pros^{V1}Gal4$ (J. de Navascués); *GS5961* (B. Ohlstein); *UAS-HepWT* (H. Jasper); *esgGAL4, NRE-GAL80*, $tub\text{-}GAL80^{ts}$*; NRE-GAL4* (B. Edgar). We generated transgenic lines with *tubGal80* inserted at sites 5B8 and 19E7 (see Vector construction below) by injection at Bestgene Inc. The *Notch* duplication NiGFP (Couturier et al., 2012) was backcrossed for 8 generations into both the w^{1118} or *Canton-S* backgrounds for Figure 2H.

Immunofluorescence

The following antibodies were used: mouse anti-Delta ECD C594.9B [ascites, 1/2000, Developmental Studies Hybridoma Bank (DSHB)]; mouse anti-Pros MR1A-c (1/1000; DSHB); chicken anti-GFP (1/4000, Abcam); goat anti-β-Gal (1/1000, Biogenesis).

Statistical analysis

For statistical analysis Fisher's exact test (two-tailed, for contingency tables) or t-test (unpaired, for differences between means) was calculated with GraphPadPrism. Significant p values were reported as follows: * $p<0.05$, *** $p<0.001$; **** $p<0.0001$, n.s.=not significant.

Ecc15, paraquat and bleomycin treatment

Adult *Canton-S* flies were treated for 48 hours on filter paper soaked with 5% sucrose covering normal food and either a 1:1 mix of OD200 *Ecc15* culture, 5 mM paraquat, 20 µg/ml bleomycin or sucrose alone. Treatment was repeated 2X per week for 4 weeks, followed by a 1-week-recovery before dissection at 5 weeks. Proliferation response was assayed by phospho-histone 3 staining 48 hours after treatment in young 1-2-week-old flies.

Vector construction

Amplification of the *GAL80* gene and the *SV40* terminator sequence were done from *tubGAL80* fly genomic DNA and from Addgene plasmid 24367, respectively. The DNA fragments were then cloned by Gibson assembly into the KpnI/BamHI site of Addgene plasmid 24352, downstream of the alpha tubulin promoter. Our sequencing revealed that this promoter is shortened on its 5' end by 142nt compared to a published sequence. The tubulin promoter region from KpnI to the start codon, including the CAAC Kozak sequence, was introduced in the forward primer. The *attB* site from pACMAN was then cloned within NdeI site to enable targeted insertion. All were sequenced. This plasmids were injected by Bestgene Inc. in strains 9753 and 9276 for *attP* insertion sites at 5B8, 19E7, respectively.

DNA FISH

Probe preparation: 5 non-overlapping 2kb genomic fragments (covering 10kb of the *Notch* locus) were PCR-amplified with following primer pairs:
AGGATGGCCCCAGCGGGA/ TGCGGCACAACACAGCGT;
CAGCTGCAACCGAAGAGCGT/ GCCATCGTCGGCGGCGT;
CGACGCCGCCGACGA/ AGTCGGCCTGGCCAAGAACA;
GTCGCCGATCGTGTGCTCGT/ CCGTTTTCCCCCGCCGGT;
TGGCAGCTGTGGCGGGG/ GCCACGTGCAACCCAAAAGG.

DNA was purified and pooled for probe labeling. Probes were labeled using the FISH Tag DNA Red Kit (Molecular Probes) according to the manufacturer's instructions.

FISH labeling: *Pros^{V1}Gal4 UAS-2XGFP* males were dissected to visually identify midguts containing neoplasias. Midguts were fixed in PBS, 4% paraformaldehyde for 30 minutes followed by 2 brief washes with PBS. Tissues were then washed once in 2XSSCT (0.3 M NaCl, 0.03 M NaCitrate, 0.1% Tween-20) and twice with 2XSSCT, 50% formamide, followed by prehybridization for 5 minutes at 92°C in 2XSSCT, 50% formamide. Probes were denatured in the hybridization buffer (2XSSCT, 50% formamide, 15% dextran sulfate, 0.5 mg/ml salmon sperm DNA) for 5 minutes at 92°C and tissue hybridization was performed overnight at 37°C. Midguts were then washed with 2XSSCT twice at 60°C and twice at room temperature and stained with DAPI to visualize nuclei.

Targeted *Notch* sequencing

Tissue isolation and target amplification: *Pros^{V1}Gal4 UAS-2XGFP* males were used to visually identify midguts containing neoplasias. The midgut region containing an estimated 40-80% neoplastic cells was manually dissected together with the neighboring control gut tissue as well as the fly head. Genomic DNA was isolated using QIAamp DNA MicroKit (Qiagen) according to the manufacturer's instructions. A 44.7kb genomic region containing the *Notch* locus was amplified with PrimeStarGXL Polymerase (Takara) in four partially overlapping fragments (overlaps of 300-1000bp), with the following primer pairs:
GAGCACTAAGAATGTGACTGCTTTCGTTTGT/ ATGCATCCGCGAGATATGGCTACTAATCAAT;
GGCCGACTCTGGCCTATCCCTTTCTCGTTC/ TTCCATGCGCAGGTGCAACACGCCCGAATA;
CCACAAACAACAGCTGGAAATGGATGGGATGGGA/ GCCATCGATGCAGGTTCCTCCGTTCTGGCA;
CTGTTTATGCACTTCATTGCACTGTGGGT/ GGCCGCCCAATATTGATTGGTATTTCTGTTA.
Equimolar amounts of purified fragments were pooled for sequencing.

Library preparation: Library preparation was performed with 100ng of pooled DNA using NEBNext® Fast DNA Fragmentation & Library Prep Set for Ion Torrent, according to the manufacturer's instructions with the following modifications. Decreased amounts of 0.5 µl of Ion Xpress Barcode and 0.5 µl of Ion P1 adaptor were used. The samples were processed simultaneously and multiplexed (with different barcodes). A size selection was set to 200bp using Agencourt® AMPure® XP beads, with a ratio of 0.7X and 0.15X respectively for the 1st and the 2nd bead selection. Library amplification was with 8 PCR cycles after which the PCR products were purified using Agencourt® AMPure® XP beads, with a ratio of 1X. The library quality was assessed on a Bioanalyzer (Agilent Technologies). An average of 376bp length and 2.6ng/µl concentration were obtained. Based on the molarity, the libraries were diluted and pooled together, at the concentration required for Ion Torrent sequencing.

Sequencing: Emulsion PCR was performed with the Ion PGM™ Template OT2 400 Kit, according to the manufacturer's instructions. The Ion PGM™ 200 sequencing kit and a 318 chip were used to sequence the 17 libraries with 500 flows set up on the Ion Torrent PGM. 1200X coverage was obtained with an average of 300,000 reads per library sample.

Sequencing data analysis of IonTorrent data

Using the standalone package of the PGM raw reads were aligned on the *Drosophila melanogaster* genome R5.54 and variant calling was performed on aligned reads using the Torrent Variant Caller (v4.0 Life Technologies). PGM quality filtering was applied.

In order to highlight long deletions, coverage over the sequenced region was analyzed. We used the DepthOfCoverage function from the Genome Analysis Toolkit (GATK v.1.6-5) software package, with mapping quality filtering of 8 and base quality filtering of 17 based on Life recommendations. Then, normalization was performed for each amplicon and amplicon overlap separately. For the global coverage report normalized coverage ratio vs. head control sample was plotted.

Whole-genome Paired End Sequencing

DNA was extracted from neoplasias (marked by *ProsGal4 UAS-GFP*), adjacent tissue and head of the same fly using QIAamp DNA MicroKit (Qiagen) according to the manufacturer's instructions. The DNA library was prepared using the DNA Nano protocol from Illumina and 2X125 bp paired-end Illumina Hi-Seq was performed by Fasteris, SA (Geneva, CHE). An average sequencing depth of 44X was achieved after duplicate read removal. Sequencing reads were mapped to *Drosophila melanogaster* genome version 5.41 by Fasteris, SA.

Analysis of Whole-genome sequencing data

CNV was assessed using CONTROL-FREEC (Boeva et al., 2012; Boeva et al., 2011). Genome regions with detected loss were visually inspected to discriminate between true copy number losses and those detected due to artifacts associated with multiply mapped sequences such as transposable elements. Loss of coverage of the *Notch* region was found and aberrant reads and split reads sequences were inspected at the location of copy number alterations using the Integrated Genomics Viewer (IGV).

For neoplasia 1 from Figure 6C, the following 8 aberrant reads supported the rearranged chromosome junction A-C (shown in Figure 6C), 5 of which contained split-reads (in bold):

HWID00405:129:C6KNAANXX:3:1304:9674:45144
HWID00405:129:C6KNAANXX:3:1308:4350:11557
HWI-D00405:129:C6KNAANXX:3:1105:11347:24760
HWI-D00405:129:C6KNAANXX:3:1207:10336:14185
HWI-D00405:129:C6KNAANXX:3:2114:7629:72645
HWI-D00405:129:C6KNAANXX:4:1111:14749:101018
HWI-D00405:129:C6KNAANXX:3:2108:19951:13460
HWI-D00405:129:C6KNAANXX:4:1303:13513:55886

The following 11 aberrant reads supported the rearranged chromosome junction inverted C-C (shown in Figure 6C), 5 of which contained split-reads (in bold):

HWI-D00405:129:C6KNAANXX:3:1216:5645:56052
HWI-D00405:129:C6KNAANXX:4:2101:5127:83936
HWI-D00405:129:C6KNAANXX:3:2309:2360:68703
HWI-D00405:129:C6KNAANXX:4:2202:4765:60335
HWI-D00405:129:C6KNAANXX:4:2313:13503:12468
HWI-D00405:129:C6KNAANXX:3:2314:18686:47690
HWI-D00405:129:C6KNAANXX:3:2316:15458:29704
HWI-D00405:129:C6KNAANXX:4:1309:8429:11556
HWI-D00405:129:C6KNAANXX:4:1108:6809:58188
HWI-D00405:129:C6KNAANXX:3:1201:12306:57632
HWI-D00405:129:C6KNAANXX:3:2313:4430:46505

The large inversion junction A-C detected in neoplasia 1 was PCR-verified using the following primer pair: TGCAAGCTGTAATTCAATTAAGGGG/ ACAGTTTGTACTCGATAAAAGTGG. Neither adjacent intestine sample nor head sample from the same fly had aberrant reads or split-reads supporting this rearrangement.

For neoplasia 2 from Figure 6D, the following 3 aberrant reads supported the rearranged chromosome junction A-C (shown in Figure 6D), all of which contained split-reads (in bold):

HWI-D00405:129:C6KNAANXX:3:2109:7116:27834
HWI-D00405:129:C6KNAANXX:4:2209:21077:11962
HWI-D00405:129:C6KNAANXX:4:2215:14766:15070

The following 8 aberrant reads supported the rearranged chromosome junction inverted C-C (shown in Figure 6D), 6 of which contained split-reads (in bold):

HWI-D00405:129:C6KNAANXX:3:1203:18655:76971
HWI-D00405:129:C6KNAANXX:4:2216:19323:12818

HWI-D00405:129:C6KNAANXX:3:1116:14016:15143
HWI-D00405:129:C6KNAANXX:4:1116:18911:7352
HWI-D00405:129:C6KNAANXX:4:2105:19433:75376
HWI-D00405:129:C6KNAANXX:3:2311:13758:27685
HWI-D00405:129:C6KNAANXX:4:1105:3773:50450
HWI-D00405:129:C6KNAANXX:3:1305:18582:50230

The adjacent tissue showed 1 aberrant read supporting an A-C junction and 3 aberrant reads supporting a C-C junction, with 1 split-read. This was very likely due to contaminating neoplastic cells as there was no loss of mapped read coverage spanning this region. In addition, the head control from this fly showed no evidence of a rearrangement or contamination.

Formally, in both neoplastic samples, the orientation of the non-duplicated sequences adjacent to the breakpoints of the junctions of inverted C-C could not be determined as they could either be part of the inverted segment or the segment in the correct orientation.

To build the sequencing coverage plot in Figure 6B and S4, coverage for each sample was determined using genomeCoverageBed from bedtools2 packaging (version 2.22.1). The plot was then built in R after normalizing each sample for the number of mapped reads. The positions identified in this plot correspond to the positions called by Control-FREEC.
